# Grape Seed Proanthocyanidins Protect Against Diabetic Retinopathy in Mice

**DOI:** 10.1002/fsn3.71337

**Published:** 2025-12-16

**Authors:** Linlin Li, Lili Tian, Yinghua Zhang, Jie Qin

**Affiliations:** ^1^ Department of Ophthalmology People's Hospital of Rizhao, Jining Medical University Rizhao Shandong China; ^2^ Department of Ophthalmology Rizhao Central Hospital Rizhao Shandong China

**Keywords:** diabetic retinopathy, grape seed proanthocyanidins, mice, Nrf2, oxidative stress

## Abstract

Grape seed proanthocyanidins (GSP), well‐known dietary nutrients for public health, have been reported to alleviate diabetic retinopathy, but the underlying mechanisms remain largely unknown. Eight‐week‐old C57BL/6 mice were used. They were fed a high‐fat diet and intraperitoneally injected with 50 mg/kg streptozotocin for five consecutive days to induce diabetes. One week later, 500 mg/kg GSP was given as a dietary supplement for 12 consecutive weeks in diabetic mice. Retinal degeneration, oxidative stress, and nuclear factor erythroid 2‐related factor 2 (Nrf2) signaling pathway were assessed. GSP significantly reduced body weight and fasting blood glucose in diabetic mice. Diabetic retinopathy‐impaired thickness of retina was also increased by GSP supplementation. Moreover, the expressions of vascular endothelial growth factor and glial fibrillary acidic protein induced by diabetic retinopathy were remarkably reduced by GSP. Furthermore, diabetic retinopathy‐provoked oxidative stress, including over‐production of reactive oxygen species and malondialdehyde, and attenuated activities of superoxide dismutase and catalase were all restored by GSP. Additionally, GSP also activated the Nrf2 signaling pathway, which was inhibited by diabetic retinopathy. These findings demonstrate that GSP ameliorates diabetic retinopathy by reducing oxidative stress, in which the Nrf2 signaling pathway is likely involved. This suggests that GSP might serve as a potential retina‐protective candidate for diabetic retinopathy.

## Graphical abstract

Grape seed proanthocyanidins (GSP) alleviated diabetic retinopathy in mice by reducing retinal degeneration, oxidative stress, and blood glucose levels. GSP restored antioxidant enzyme activities, decreased reactive oxygen species and malondialdehyde, and suppressed VEGF and GFAP expression. Additionally, GSP activated the Nrf2 signaling pathway, which was inhibited in diabetic conditions. These results suggest that GSP mitigates diabetic retinopathy through oxidative stress reduction and Nrf2 activation, indicating its potential as a protective dietary supplement for retinal health.

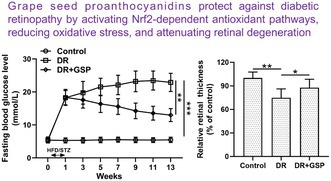

## 1 Introduction

Diabetic retinopathy is a leading cause of preventable blindness worldwide, resulting from progressive damage to the retinal microvasculature in patients with diabetes mellitus (Cheung et al. 2010). Despite advances in clinical care, current strategies are largely limited to glycemic control and invasive interventions, such as laser therapy or anti‐vascular endothelial growth factor (VEGF) injections, which are not curative. At least 90% of new cases could be prevented with earlier interventions, highlighting the urgent need for novel preventive and therapeutic approaches.

Oxidative stress has emerged as a central pathogenic mechanism in diabetic retinopathy, linking hyperglycemia to vascular leakage, neurodegeneration, and inflammatory responses (Hussain et al. 2023; Kang and Yang 2020). Consequently, antioxidant‐based interventions are increasingly recognized as promising candidates for attenuating diabetic retinopathy progression.

Grape seed proanthocyanidins (GSP) are polyphenolic compounds first extracted from grape seeds and pine bark in 1947. They have been shown to possess antioxidant, neuroprotective, cardioprotective, and immunomodulatory properties (de la Iglesia et al. 2010; Rauf et al. 2019). In ocular diseases, GSP has demonstrated protective effects by reducing reactive oxygen species (ROS), preserving mitochondrial function, and attenuating neuronal injury (Li et al. 2020). Clinical and preclinical evidence suggests their potential benefit in diabetic retinopathy (Li et al. 2008; Moon et al. 2019). However, the precise molecular mechanisms underlying their protective effects in diabetic retinopathy remain largely undefined.

Here, we hypothesized that GSP protects against diabetic retinopathy by attenuating oxidative stress through activation of the nuclear factor erythroid 2‐related factor 2 (Nrf2) signaling pathway. Using a streptozotocin (STZ)‐induced diabetic mouse model, we investigated whether GSP supplementation could restore retinal integrity, modulate oxidative stress markers, and activate endogenous antioxidant responses.

## 2 Materials and Methods

### 2.1 Diabetic Retinopathy Mouse Model

Eight‐week‐old C57BL/6 male mice were used in this study. They were fed a high‐fat diet (60 kcal% fat, 20 kcal% carbohydrate, 20 kcal% protein, with a total energy of 5.24 kcal/g, #D12492; Research Diets Inc., USA) and intraperitoneally injected with 50 mg/kg STZ daily for five consecutive days from Day 1. Saline‐injected mice were considered control mice. On Day 6, mice with fasting blood glucose over 16.7 mmol/L were selected as diabetic mice.

After STZ injection, diabetic mice were continuously maintained on a high‐fat diet for 12 weeks. For GSP treatment, GSP was administered as a dietary supplement by mixing powdered GSP with high‐fat diet pellets. The mixing process followed our previously published protocol (Li et al. 2020). Briefly, GSP powder was first ground with 10% of the diet until the particle size was < 100 μm to avoid aggregation, then blended with the remaining feed using a three‐dimensional mixer, followed by sieving and pelletizing to match the 2 mm pellet size of the standard diet. The daily intake of GSP was calculated based on the formula *D* = *d* × *t*/*W* (*d*: individual dose; *t*: administration frequency; *W*: average daily food intake). Considering the average food consumption of diabetic mice (~4 g/mouse/day), the final daily intake corresponded to 500 mg/kg body weight. Mice were sacrificed at week 13, and retinal tissues were collected for analysis. The study was approved by the Laboratory Animal Ethics Committee of Jining Medical University (#JNMC‐2023‐DW‐061).

The mice were divided into three experimental groups: Control group: non‐diabetic mice; DR group: diabetic mice, induced as described above; DR + GSP group: diabetic mice treated with GSP.

### 2.2 qRT‐PCR

Total mRNA was extracted from retina, and qRT‐PCR was performed as previously described (Liu et al. 2021). The gene expressions were normalized to GAPDH. The examined mRNA expression of the genes included vascular endothelial growth factor (*VEGF*), glial fibrillary acidic protein (*GFAP*), and *Nrf2*.

The sequences of primers were as below:

- *VEGF*: Forward: TTTGGCAAATACAACCCTTCAGA; Reverse: GCTCCAGTATCATTTCCAACCA.
- *GFAP*: Forward: CGGAGACGCATCACCTCTG; Reverse: TGGAGGAGTCATTCGAGACAA.
- *Nrf2*: Forward: CTTTAGTCAGCGACAGAAGGAC; Reverse: AGGCATCTTGTTTGGGAATGTG.
- *GAPDH*: Forward: TTCACCACCATGGAGAAGGC; Reverse: GGCATGGACTGTGGTCATGA.

### 2.3 Hematoxylin and Eosin (H&E) Staining

Eyeballs collected from different groups of mice were fixed in 4% formalin, embedded in paraffin, and cut into 5 μm sections at the same orientation through the optic nerve head. H&E staining was performed as previously described (Cheng et al. 2019). The thickness of retinal cross‐sections was quantified at a constant distance from the optic nerve head using Image‐Pro Plus 6.0 analysis software.

### 2.4 Western Blot

Western blot was performed as previously described to analyze the protein levels in the retina (Liu et al. 2012). Densitometry analyses of the bands were performed using Image J software. Proteins were normalized to GAPDH.

### 2.5 Dihydroethidium (DHE) Staining

After collecting from mice, retinal tissues were immediately frozen in optimal cutting temperature compound (Sakura Finetek, USA) and cut into 10 μm serial cryosections. Sections were incubated with 5 mM DHE for 20 min at 37°C; then fluorescence was determined using a fluorescence microscope. The fluorescence density was analyzed using ImageJ software (Li et al. 2020).

### 2.6 Measurement of Oxidative Stress

The levels of Malondialdehyde were measured using a lipid peroxidation malondialdehyde (MDA) assay kit (#S0131M; Beyotime). The activities of catalase (CAT) and superoxide dismutase (SOD) were determined with the catalase assay kit (#CAT100; Sigma‐Aldrich, USA) and the superoxide dismutase assay kit (#S0101M; Beyotime) respectively according to the manufacturers' instructions (Tu et al. 2021).

### 2.7 Statistical Analysis

Data were presented as means ± standard deviation (SD). One‐way ANOVA followed Dunn's multiple comparisons test or two‐way ANOVA followed Tukey's multiple comparisons test was used for statistical analysis.

## 3 Results

### 3.1 Effects of GSP on Body Weight and Fasting Blood Glucose

**FIGURE 1:**
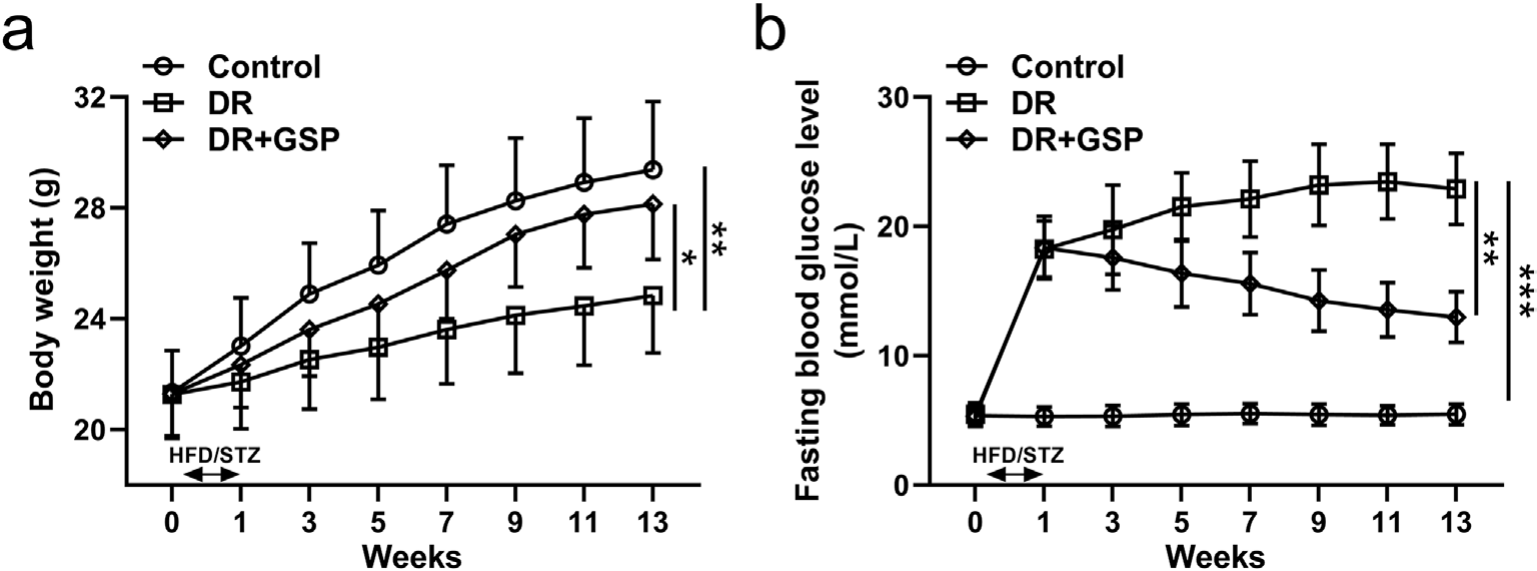
The effects of GSP on the body weight (a) and fasting blood glucose level (b) in the mice with diabetic retinopathy (DR). Data are presented as means ± SD from 12 mice in each group. *p < 0.05, **p < 0.01, ***p < 0.001 between the indicated groups. Two‐way ANOVA followed by Tukey's multiple comparisons test.

We used three different doses of GSP (125, 250, and 500 mg/kg) in our previous study (Li et al. 2020), therefore, based on our previous data, here we only selected the 500 mg/kg dose of GSP. Compared to control mice, STZ‐induced diabetic mice displayed remarkably increased body weight and fasting blood glucose, which could be significantly reduced by GSP supplementation (Figure 1a,b). These data suggested the anti‐diabetic effects of GSP.

### 3.2 GSP Increases the Thickness of the Retinal Tissue in Diabetic Mice

**FIGURE 2:**
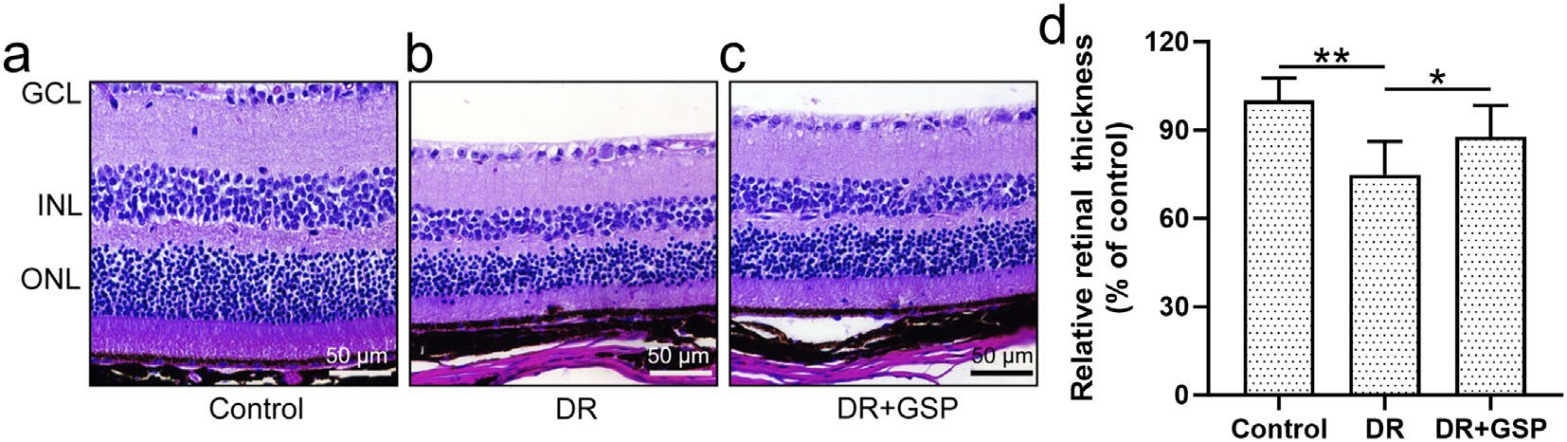
The effects of GSP on the thickness of the retinal tissue in the mice with diabetic retinopathy (DR). Representative hematoxylin and eosin staining of retinal cross‐sections in the experimental mice, including (a) control, (b) DR, and (c) DR + GSP groups. (d) The relative retinal tissue thickness in these groups. Data were presented as means ± SD from six mice in each group. *p < 0.05, **p < 0.01 between the indicated groups. One‐way ANOVA followed Dunn's multiple comparisons test. Retinal layers are labeled: Ganglion cell layer (GCL), inner nuclear layer (INL), outer nuclear layer (ONL).

H&E staining showed that diabetic mice (Figure 2b) had a thinned thickness of retinal tissue in comparison with control mice (Figure 2a), which indicated the successful establishment of diabetic retinopathy. As expected, impaired thickness of retinal tissue was also increased by the supplement of GSP (Figure 2c,d). Altogether, GSP could ameliorate retinal degeneration in diabetic mice.

### 3.3 Effects of GSP on mRNA Expressions of VEGF, GFAP and Nrf2 in Diabetic Mice

**FIGURE 3:**
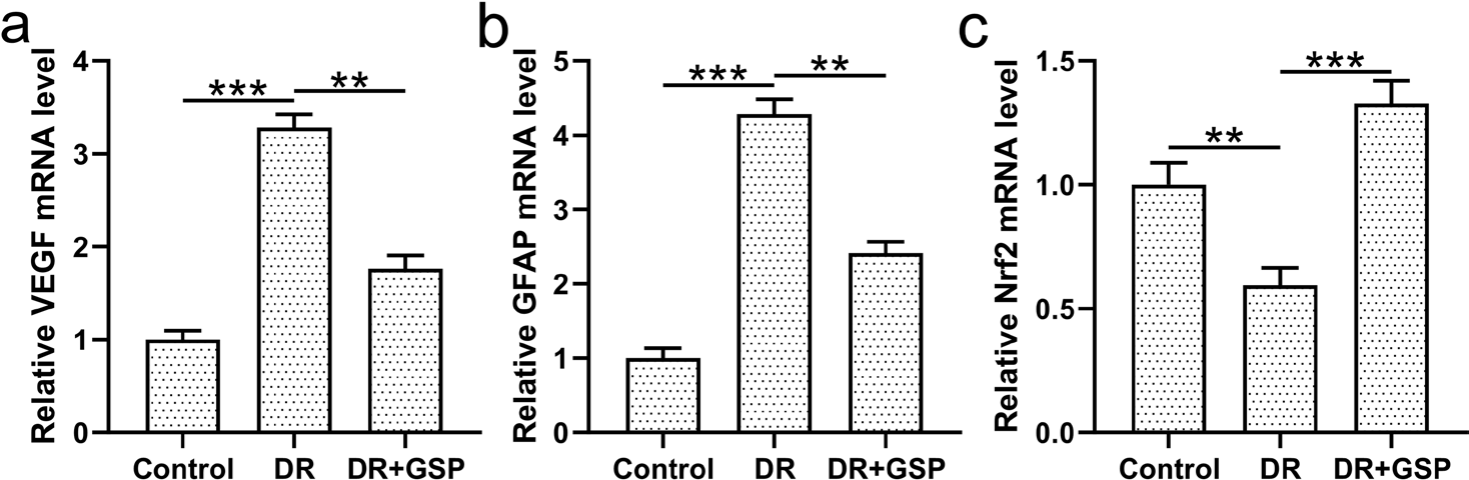
The effects of GSP treatment on mRNA expressions of key genes in the retina of mice with diabetic retinopathy (DR). (a) Relative *VEGF* mRNA level, (b) relative *GFAP* mRNA level, and (c) relative *Nrf2* mRNA level in retinal tissues from control, DR, and DR + GSP groups. qRT‐PCR was performed with *GAPDH* as the internal control. *n* = 3 from eight mice in each group. Data were presented as means ± SD. **p < 0.01, ***p < 0.001 between the indicated groups. One‐way ANOVA followed Dunn's multiple comparisons test.

Previous publications indicated the importance of VEGF and GFAP in the pathogenesis of diabetic retinopathy; therefore, we also explored GSP's effect on VEGF and GFAP in the development of diabetic retinopathy (Cheng et al. 2019; Tu et al. 2021). Compared to control mice, mRNA expressions of both *VEGF* and *GFAP* were upregulated in the retinal tissues of diabetic retinopathy mice, which were significantly downregulated by GSP treatment (Figure 3a,b). Moreover, mRNA expression of *Nrf2* was downregulated in the retinal tissues of diabetic retinopathy mice, while GSP treatment significantly reversed this decrease (Figure 3c). These data suggested that VEGF and GFAP might be involved in the protective effect of GSP on diabetic retinopathy.

### 3.4 Effects of GSP on Protein Expressions of VEGF, GFAP and Nrf2 in Diabetic Mice

**FIGURE 4:**
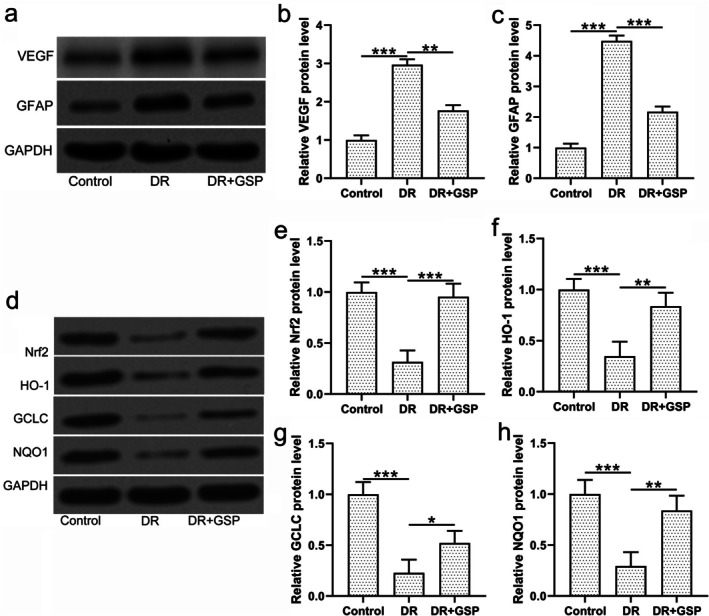
The effects of GSP treatment on protein expressions in the mice with diabetic retinopathy (DR), profiling by western blotting. (a) Representative Western blots of VEGF and GFAP in the retinas of experimental mice. Relative protein expression of (b) VEGF and (c) GFAP. (d) Representative western blots of Nrf2, HO‐1, GCLC, and NQO1 in the retinas of experimental mice. Relative protein expression of (e) Nrf2, (f) HO‐1, (g) GCLC, and (h) NQO1. The expressions were normalized to their corresponding GAPDH control. *n* = 3 from eight mice in each group. *p < 0.05, **p < 0.01, ***p < 0.001 between the indicated groups. One‐way ANOVA followed Dunn's multiple comparisons test.

Furthermore, the protein expressions of VEGF, GFAP, and Nrf2 were evaluated by Western blot assays. It was found that both VEGF and GFAP protein expressions were significantly upregulated in the retinal tissues of diabetic retinopathy mice when compared to control mice, while GSP treatment successfully and significantly downregulated these increases (Figure 4a–c). Considering that GSP was reported to attenuate immunotoxicity and oxidative stress through the Nrf2 signaling pathway (Rajput et al. 2019), the Nrf2 signaling pathway was also evaluated. It was revealed that GSP significantly restored the protein levels of Nrf2 reduced by diabetic retinopathy in the mice (Figure 4d,e). Additionally, the protein expressions of heme oxygenase‐1 (HO‐1), glutamate‐cysteine ligase catalytic subunit (GCLC), and NAD(P)H dehydrogenase (quinone 1) (NQO1) were all significantly reduced in mice with diabetic retinopathy, and all were significantly restored by the supplement of GSP (Figure 4d,f–h). These data revealed that GSP activated the Nrf2 signaling pathway in the diabetic mice.

### 3.5 Effects of GSP on Oxidative Stress Induced by Diabetic Retinopathy

**FIGURE 5:**
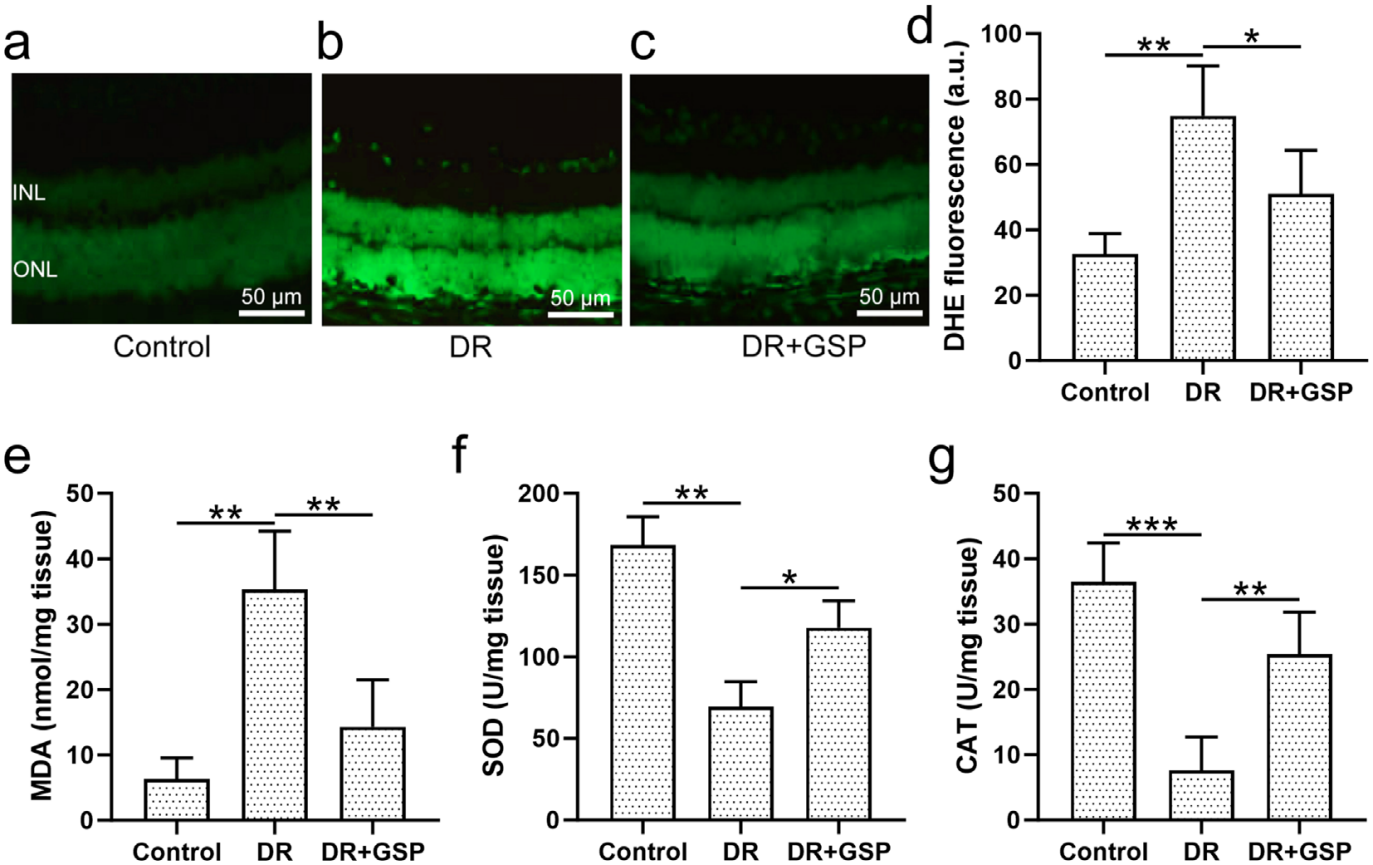
The effects of GSP on retinal oxidative stress in mice with diabetic retinopathy (DR). Representative DHE staining of retinal slices from (a) control, (b) DR, and (c) DR + GSP groups. (d) Quantification of DHE staining in these groups. (e) MDA production, (f) SOD activity, and (g) CAT levels in the retina of mice were analyzed by ELISA kits. Data were presented as means ± SD from six mice in each group. *p < 0.05, **p < 0.01, ***p < 0.001 between the indicated groups. One‐way ANOVA followed Dunn's multiple comparisons test.

Oxidative stress was implicated in the pathogenesis of diabetic retinopathy (Calderon et al. 2017), therefore, we assessed the anti‐oxidative effect of GSP in the development of diabetic retinopathy. We first evaluated ROS levels in the retinal tissue from different groups of mice. DHE staining indicated that, compared to control mice (Figure 5a,d), ROS production was increased in diabetic mice (Figure 5b,d) and restored by the administration of GSP (Figure 5c,d). Moreover, we also measured the oxidative stress marker MDA in the retina, and diabetic retinopathy‐induced MDA expressions in the retina were significantly reduced by GSP (Figure 5e). In addition, the antioxidant enzymes, including SOD and CAT, which are also markers of oxidative stress, were evaluated as well. The enzymatic activities of SOD (Figure 5f) and CAT (Figure 5g), which were diminished in diabetic mice, were provoked by the supplement of GSP. Thus, GSP effectively reduced diabetic retinopathy‐induced oxidative stress in the retina of diabetic mice.

## 4 Discussion

GSP, which contains polymers or polyhydroxyflavan oligomers, has attracted wide attention as supplementary nutrients, due to their beneficial health properties. There might be a two‐way relationship between GSP and gut microbiota, contributing to the pharmacological effect of GSP, such as anti‐oxidant, anti‐obesity, anti‐diabetic, anti‐microbial, anti‐osteoarthritis, anti‐neurodegenerative, anti‐cancer, and cardiovascular‐ and eye‐protective properties (Rauf et al. 2019; Rodriguez‐Perez et al. 2019; Shi et al. 2003; Unusan 2020). In the present study, we demonstrate that GSP supplementation significantly alleviates diabetic retinopathy pathology, as evidenced by improved retinal thickness, reduced VEGF and GFAP expression, mitigation of oxidative stress, and activation of the Nrf2 pathway. These findings extend our previous observations in glaucoma (Li et al. 2020) and provide new mechanistic insights into GSP's actions in diabetic retinopathy.

Our previous study revealed that GSP effectively protected retinal ganglion cells by inhibiting oxidative stress and mitochondrial dysfunction in glaucomatous DBA/2D (D2) mice (Li et al. 2020). Therefore, in the present project, our group continued to explore the protective effects of GSP on eye diseases, as is diabetic retinopathy.

A body of publications indicated that GSP effectively attenuated diabetic retinopathy in multiple aspects. For example, 1‐year oral GSP treatment attenuated the severity of hard exudates in patients with non‐proliferative diabetic retinopathy (Moon et al. 2019). Furthermore, GSP significantly reduced advanced glycation end products (AGEs) in STZ‐induced diabetic retinopathy rats. However, the underlying mechanism remains largely unknown. Therefore, in our study, we further explored the protective effect of GSP as an antioxidant on diabetic retinopathy.

Oxidative stress is a cytopathic consequence of reactive oxygen species over‐production and the inhibition of the antioxidant defense system, which is indicated in the pathogenesis of a variety of diseases, including diabetes and its complications (Calderon et al. 2017). Therefore, oxidative stress was indicated to be tightly associated with the pathogenesis of diabetic retinopathy (Kowluru and Chan 2007; Li et al. 2008; Madsen‐Bouterse and Kowluru 2008; Pan et al. 2008). Thus, as a strong antioxidant, we speculated that GSP could ameliorate diabetic retinopathy by reducing oxidative stress. Our data confirmed our hypothesis that GSP significantly improved diabetic retinopathy by reducing ROS production and oxidative stress in the retina of diabetic mice.

In addition, we further explored the pathophysiological mechanism of GSP on diabetic retinopathy. The Nrf2 signaling pathway was identified to have an anti‐oxidative effect (Jiang et al. 2016), and it is also suggested to mediate the protective effect of GSP on oxidative stress (Rajput et al. 2019). Therefore, here we evaluated the role of Nrf2 in GSP‐improved diabetic retinopathy, and our data confirmed that Nrf2 expressions were reduced in diabetic retinopathy.

Mice were significantly increased by GSP, which suggested that GSP activated Nrf2. The activation of the Nrf2 signaling pathway protects against damage from oxidative stress, consequently activating the antioxidative system as well as mediating the expressions of several intracellular antioxidant genes. Therefore, we also measured Nrf2's target genes, including *HO‐1*, *NQO1*, and *GCLC*. Instead of evaluating mRNA levels of these genes, we directly assessed their protein levels. These proteins are the important components of the redox system, and they exert their cytoprotective resistance effect to oxidative stress. These proteins were downregulated in the retina of mice with diabetic retinopathy, and GSP remarkably upregulated these proteins to activate the endogenous defense system to oxidative stress.

We selected a dose of 500 mg/kg GSP based on our prior work in ocular disease models, where dose–response testing (125, 250, and 500 mg/kg) showed the most consistent protection at the highest dose (Li et al. 2020). In the current study, our aim was to maximize mechanistic sensitivity rather than conduct a new dose–response analysis. While this approach limits translational extrapolation, the selected dose is consistent with rodent antioxidant studies and may be clinically relevant with concentrated GSP formulations. Future research incorporating graded dosing and pharmacokinetic studies will be essential to define optimal and clinically translatable regimens.

Nevertheless, several limitations of the present study should be acknowledged more explicitly. First, only male mice were included, and potential sex‐specific differences in diabetic retinopathy pathogenesis and GSP responsiveness were not evaluated. Second, we employed a single GSP dosage (500 mg/kg) without testing lower or intermediate doses, which restricts dose–response interpretation. Third, we focused primarily on morphological and molecular markers; functional visual outcomes, such as electroretinography (ERG), were not assessed and should be incorporated in future work to strengthen translational relevance.

Looking ahead, future studies could explore pathways for clinical translation, such as assessing GSP in combination with established diabetic treatments (e.g., glycemic control agents, anti‐VEGF therapy, or other antioxidants). Given that GSP exerts multi‐target actions—including suppression of oxidative stress, inflammation, and pathological angiogenesis—it may complement single‐target therapies and enhance overall treatment efficacy. Moreover, longitudinal and sex‐balanced animal studies, coupled with functional visual assessments and eventual clinical trials, will be essential to fully establish the therapeutic potential of GSP in diabetic retinopathy.

## 5 Conclusions

This study provides evidence that GSP protects against diabetic retinopathy by activating Nrf2‐dependent antioxidant pathways, reducing oxidative stress, and attenuating retinal degeneration. These findings support the potential of GSP as an adjunctive therapeutic approach for diabetic retinopathy and justify further preclinical dose‐optimization studies and eventual clinical trials.

## Author Contributions

**Linlin Li:** data curation (lead), validation (lead), writing – original draft (lead), writing – review and editing (lead). **Lili Tian:** data curation (supporting), validation (supporting), writing – original draft (supporting), writing – review and editing (supporting). **Yinghua Zhang:** data curation (supporting), validation (supporting), writing – original draft (supporting), writing – review and editing (supporting). **Jie Qin:** data curation (supporting), supervision (lead), conception (lead), writing – original draft (supporting), writing – review and editing (supporting).

## Funding

This work was supported by the Project of Natural Science Foundation of Rizhao City (#RZ2021ZR23).

## Ethics Statement

The study was approved by the Laboratory Animal Ethics Committee of Jining Medical University (#JNMC‐2023‐DW‐061). This study was performed in strict accordance with the NIH guidelines for the care and use of laboratory animals (NIH Publication No. 85‐23 Rev. 1985).

## Consent

The authors have nothing to report.

## Conflicts of Interest

The authors declare no conflicts of interest.

## Data Availability

The raw data could be obtained upon reasonable request to the corresponding author.

## References

[fsn371337-bib-0001] Calderon, G. D. , O. H. Juarez , G. E. Hernandez , S. M. Punzo , and Z. D. De la Cruz . 2017. “Oxidative Stress and Diabetic Retinopathy: Development and Treatment.” Eye (London, England) 31, no. 8: 1122–1130. 10.1038/eye.2017.64. 28452994 PMC5558229

[fsn371337-bib-0002] Cheng, Y. , X. Yu , J. Zhang , et al. 2019. “Pancreatic Kallikrein Protects Against Diabetic Retinopathy in KK Cg‐A(y)/J and High‐Fat Diet/Streptozotocin‐Induced Mouse Models of Type 2 Diabetes.” Diabetologia 62, no. 6: 1074–1086. 10.1007/s00125-019-4838-9. 30838453 PMC6509079

[fsn371337-bib-0003] Cheung, N. , P. Mitchell , and T. Y. Wong . 2010. “Diabetic Retinopathy.” Lancet 376, no. 9735: 124–136. 10.1016/S0140-6736(09)62124-3. 20580421

[fsn371337-bib-0004] de la Iglesia, R. , F. I. Milagro , J. Campion , N. Boque , and J. A. Martinez . 2010. “Healthy Properties of Proanthocyanidins.” BioFactors 36, no. 3: 159–168. 10.1002/biof.79. 20232344

[fsn371337-bib-0005] Hussain, A. , S. Ashique , O. Afzal , et al. 2023. “A Correlation Between Oxidative Stress and Diabetic Retinopathy: An Updated Review.” Experimental Eye Research 236: 109650. 10.1016/j.exer.2023.109650. 37734426

[fsn371337-bib-0006] Jiang, Y. M. , Y. Wang , H. S. Tan , et al. 2016. “Schisandrol B Protects Against Acetaminophen‐Induced Acute Hepatotoxicity in Mice via Activation of the NRF2/ARE Signaling Pathway.” Acta Pharmacologica Sinica 37, no. 3: 382–389. 10.1038/aps.2015.120. 26806302 PMC4775844

[fsn371337-bib-0007] Kang, Q. , and C. Yang . 2020. “Oxidative Stress and Diabetic Retinopathy: Molecular Mechanisms, Pathogenetic Role and Therapeutic Implications.” Redox Biology 37: 101799. 10.1016/j.redox.2020.101799. 33248932 PMC7767789

[fsn371337-bib-0008] Kowluru, R. A. , and P. S. Chan . 2007. “Oxidative Stress and Diabetic Retinopathy.” Experimental Diabetes Research 2007: 43603. 10.1155/2007/43603. 17641741 PMC1880867

[fsn371337-bib-0009] Li, L. , X. Geng , L. Tian , D. Wang , and Q. Wang . 2020. “Grape Seed Proanthocyanidins Protect Retinal Ganglion Cells by Inhibiting Oxidative Stress and Mitochondrial Alteration.” Archives of Pharmacal Research 43, no. 10: 1056–1066. 10.1007/s12272-020-01272-9. 33078305

[fsn371337-bib-0010] Li, M. , Y. B. Ma , H. Q. Gao , et al. 2008. “A Novel Approach of Proteomics to Study the Mechanism of Action of Grape Seed Proanthocyanidin Extracts on Diabetic Retinopathy in Rats.” Chinese Medical Journal 121, no. 24: 2544–2552. https://www.ncbi.nlm.nih.gov/pubmed/19187593. 19187593

[fsn371337-bib-0011] Liu, Z. , M. R. Iyer , G. Godlewski , et al. 2021. “Functional Selectivity of a Biased Cannabinoid‐1 Receptor (CB(1)R) Antagonist.” ACS Pharmacology & Translational Science 4, no. 3: 1175–1187. 10.1021/acsptsci.1c00048. 34151207 PMC8204328

[fsn371337-bib-0012] Liu, Z. , H. Luo , L. Zhang , et al. 2012. “Hyperhomocysteinemia Exaggerates Adventitial Inflammation and Angiotensin II‐Induced Abdominal Aortic Aneurysm in Mice.” Circulation Research 111, no. 10: 1261–1273. 10.1161/CIRCRESAHA.112.270520. 22912384

[fsn371337-bib-0013] Madsen‐Bouterse, S. A. , and R. A. Kowluru . 2008. “Oxidative Stress and Diabetic Retinopathy: Pathophysiological Mechanisms and Treatment Perspectives.” Reviews in Endocrine & Metabolic Disorders 9, no. 4: 315–327. 10.1007/s11154-008-9090-4. 18654858

[fsn371337-bib-0014] Moon, S. W. , Y. U. Shin , H. Cho , S. H. Bae , H. K. Kim , and for the Mogen Study Group . 2019. “Effect of Grape Seed Proanthocyanidin Extract on Hard Exudates in Patients With Non‐Proliferative Diabetic Retinopathy.” Medicine (Baltimore) 98, no. 21: e15515. 10.1097/MD.0000000000015515. 31124931 PMC6571433

[fsn371337-bib-0015] Pan, H. Z. , H. Zhang , D. Chang , H. Li , and H. Sui . 2008. “The Change of Oxidative Stress Products in Diabetes Mellitus and Diabetic Retinopathy.” British Journal of Ophthalmology 92, no. 4: 548–551. 10.1136/bjo.2007.130542. 18369071

[fsn371337-bib-0016] Rajput, S. A. , L. Sun , N. Y. Zhang , et al. 2019. “Grape Seed Proanthocyanidin Extract Alleviates AflatoxinB1‐Induced Immunotoxicity and Oxidative Stress via Modulation of NF‐κB and Nrf2 Signaling Pathways in Broilers.” Toxins 11, no. 1: 10023. 10.3390/toxins11010023. PMC6356337 30621062

[fsn371337-bib-0017] Rauf, A. , M. Imran , T. Abu‐Izneid , et al. 2019. “Proanthocyanidins: A Comprehensive Review.” Biomedicine & Pharmacotherapy 116: 108999. 10.1016/j.biopha.2019.108999. 31146109

[fsn371337-bib-0018] Rodriguez‐Perez, C. , B. Garcia‐Villanova , E. Guerra‐Hernandez , and V. Verardo . 2019. “Grape Seeds Proanthocyanidins: An Overview of In Vivo Bioactivity in Animal Models.” Nutrients 11, no. 10: 2435. 10.3390/nu11102435. 31614852 PMC6835351

[fsn371337-bib-0019] Shi, J. , J. Yu , J. E. Pohorly , and Y. Kakuda . 2003. “Polyphenolics in Grape Seeds‐Biochemistry and Functionality.” Journal of Medicinal Food 6, no. 4: 291–299. 10.1089/109662003772519831. 14977436

[fsn371337-bib-0020] Tu, Y. , E. Song , Z. Wang , et al. 2021. “Melatonin Attenuates Oxidative Stress and Inflammation of Muller Cells in Diabetic Retinopathy via Activating the Sirt1 Pathway.” Biomedicine & Pharmacotherapy 137: 111274. 10.1016/j.biopha.2021.111274. 33517190

[fsn371337-bib-0021] Unusan, N. 2020. “Proanthocyanidins in Grape Seeds: An Updated Review of Their Health Benefits and Potential Uses in the Food Industry.” Journal of Functional Foods 67: 103861.

